# Pathways from the Superior Colliculus to the Basal Ganglia

**DOI:** 10.2174/1570159X21666230911102118

**Published:** 2023-09-11

**Authors:** Fernando Falkenburger Melleu, Newton Sabino Canteras

**Affiliations:** 1 Department of Anatomy, Institute of Biomedical Sciences, University of Sao Paulo, Sao Paulo, SP, Brazil

**Keywords:** Superior colliculus, striatum, thalamus, subthalamic nucleus, subcortical loops, behavior

## Abstract

The present work aims to review the structural organization of the mammalian superior colliculus (SC), the putative pathways connecting the SC and the basal ganglia, and their role in organizing complex behavioral output. First, we review how the complex intrinsic connections between the SC’s laminae projections allow for the construction of spatially aligned, visual-multisensory maps of the surrounding environment. Moreover, we present a summary of the sensory-motor inputs of the SC, including a description of the integration of multi-sensory inputs relevant to behavioral control. We further examine the major descending outputs toward the brainstem and spinal cord. As the central piece of this review, we provide a thorough analysis covering the putative interactions between the SC and the basal ganglia. To this end, we explore the diverse thalamic routes by which information from the SC may reach the striatum, including the pathways through the lateral posterior, parafascicular, and rostral intralaminar thalamic nuclei. We also examine the interactions between the SC and subthalamic nucleus, representing an additional pathway for the tectal modulation of the basal ganglia. Moreover, we discuss how information from the SC might also be relayed to the basal ganglia through midbrain tectonigral and tectotegmental projections directed at the substantia nigra compacta and ventrotegmental area, respectively, influencing the dopaminergic outflow to the dorsal and ventral striatum. We highlight the vast interplay between the SC and the basal ganglia and raise several missing points that warrant being addressed in future studies.

## 1 INTRODUCTION

The superior colliculus (or optic tectum in non-mammalian species) occupies the dorsal most region of the mesencephalon and is well-conserved across vertebrates [1, 2]. Although the degree of development of the optic tectum may vary in different species, its layered structure, hodology, and cell types are fairly congruent in all vertebrates [1, 2]. Classically, the superior colliculus (SC) was regarded as a mainly visual structure, being the primary way-station for fibers arising from the optic tract and, thus, involved in controlling simple visually evoked reflexes [3, 4]. In species such as birds and primates, where visual information is the predominant sensory modality conveying environmental features, the SC presents a more well-developed structure and seems to play a more prominent role in visual-guided behaviors [5-10]. The same is true for mammals with arboreal habitats, such as squirrels, tree shrews, and some new world primates that depend on visual information to explore and move around their environments [11-13].

While true that the SC receives an expressive amount of visual input directly from the retina and that such projections can be the main drivers of some of the SC’s functions, in most species [8, 10, 14-19], its layered structure presents a topographically segregated pattern of multimodal sensory inputs, as well as direct and indirect motor outputs aimed toward the reticular formation and spinal cord [5, 20-37]. In other words, the SC is involved in the processing and integrating of a plethora of sensory information, ultimately influencing motor behavior [38-40].

Furthermore, the complex intrinsic connections between the SC’s laminae and tecto-tectal projections allow for the construction of spatially aligned, visual-multisensory maps of the surrounding environment [25, 38, 41-47]. These multi-modal sensory maps then contribute to the diverse functions of the SC [48-50]. The functional roles of the SC in driving motivated behaviors can be divided into two categories: The first includes behavioral responses that are initiated and organized by the SC. These behaviors include saccadic eye movements [51], pinnae movements [52], whisker movements [53, 54], and movements of the head and limbs that orient the animals toward or away from external stimuli [55-61]. In the second category are behaviors that are organized by different brain networks but are nonetheless modulated by inputs from the SC. Thus, the SC constitutes part of other neural networks by being a significant source of information about the environment, providing other structures with multisensory maps aligned with the visual space [62-64]. This category includes behaviors such as prey capture [64-67], drinking behavior [68, 69], and innate defensive responses [70-74]. This functional segregation is a consequence of the diverse inputs and outputs integrated by the SC, as well as its layered structure and the distribution of afferents and efferents within the SC. The structural organization of the SC will be discussed in greater detail further.

Since the SC can organize and modulate motor functions, it is not surprising that the SC coordinates its activity with several basal ganglia (BG) networks [75-79]. In this regard, it is relevant that cortical projection neurons generate collateral projections to innervate both striatal and tectal targets [36]. This way, cortico-tectal and cortico-striatal projections can interact with each other through SC-BG-cortical or cortico-BG-tectal multi-synaptic pathways. Connections between the BG and the SC form re-entrant parallel closed-feedback loops that allow for adaptive and smooth collicular-driven/modulated actions. As we will see in greater detail, most of these loops are relayed from the SC to the structures of the basal ganglia through several thalamic districts [66, 78]. The SC harbors distinct thalamic projecting neurons and receives direct convergent inputs from functionally correlated cortical areas and inputs from specific domains within the reticular part of the substantia nigra (SNr), the motor output node of the basal ganglia subnetworks [36].

In the last 20 years, the interplay between the basal ganglia and the SC and how this affects behavior and motor function has been the subject of several studies. Furthermore, the somewhat recent advent of techniques such as optogenetics, chemogenetics, calcium imaging, single-unit electrophysiological recordings, and viral vector pathway tracing, coupled with the use of transgenic animals, have greatly advanced our comprehension of this topic.

The present work aims to review the structural organization of the mammalian SC and the pathways connecting the SC and the basal ganglia, as well as their role in organizing complex behavioral output. Moreover, we present a summary of the sensory afferents and motor projections of the SC, including a description of processes that allow for the integration of multi-sensory input relevant to behavioral control and interaction with structures of the basal ganglia.

In this review, we focus on rodents and present data on their SC unless otherwise specified. Furthermore, the exact neurochemical identity of most of the afferent and efferent connections of the SC is still largely unknown. However, the available data on the neurochemistry of SC connections has been recently reviewed elsewhere [80]. Therefore, we will highlight only the neurotransmitters that are known to be relevant for behavior and the interactions between the SC and the basal ganglia when appropriate.

Regarding the functional side, most recent studies investigating the behavioral role of the SC-BG pathways, except for those on oculomotor function in primates, have been conducted in rodents. Therefore, we will also present these findings in murine subjects unless otherwise stated.

Additionally, we will briefly discuss the functional significance of the SC-BG pathways in human motor and cognitive disorders. We will also touch upon investigations of animal models of neurodegenerative diseases to shed light on the potential implications of these pathways for neurological diseases.

## 2 CYTOARCHITECTONIC AND HODOLOGICAL ORGANIZATION OF THE MAMMALIAN SUPERIOR COLLICULUS

To fully appreciate the interaction between the SC and the basal ganglia, it is essential to appraise the architecture, sensory inputs, and integrative nature of the SC. Since the connections of the SC have been extensively studied and reviewed in previous works [36, 80, 81], here we present a brief summary of the SC’s organization.

**Fig. (1):**
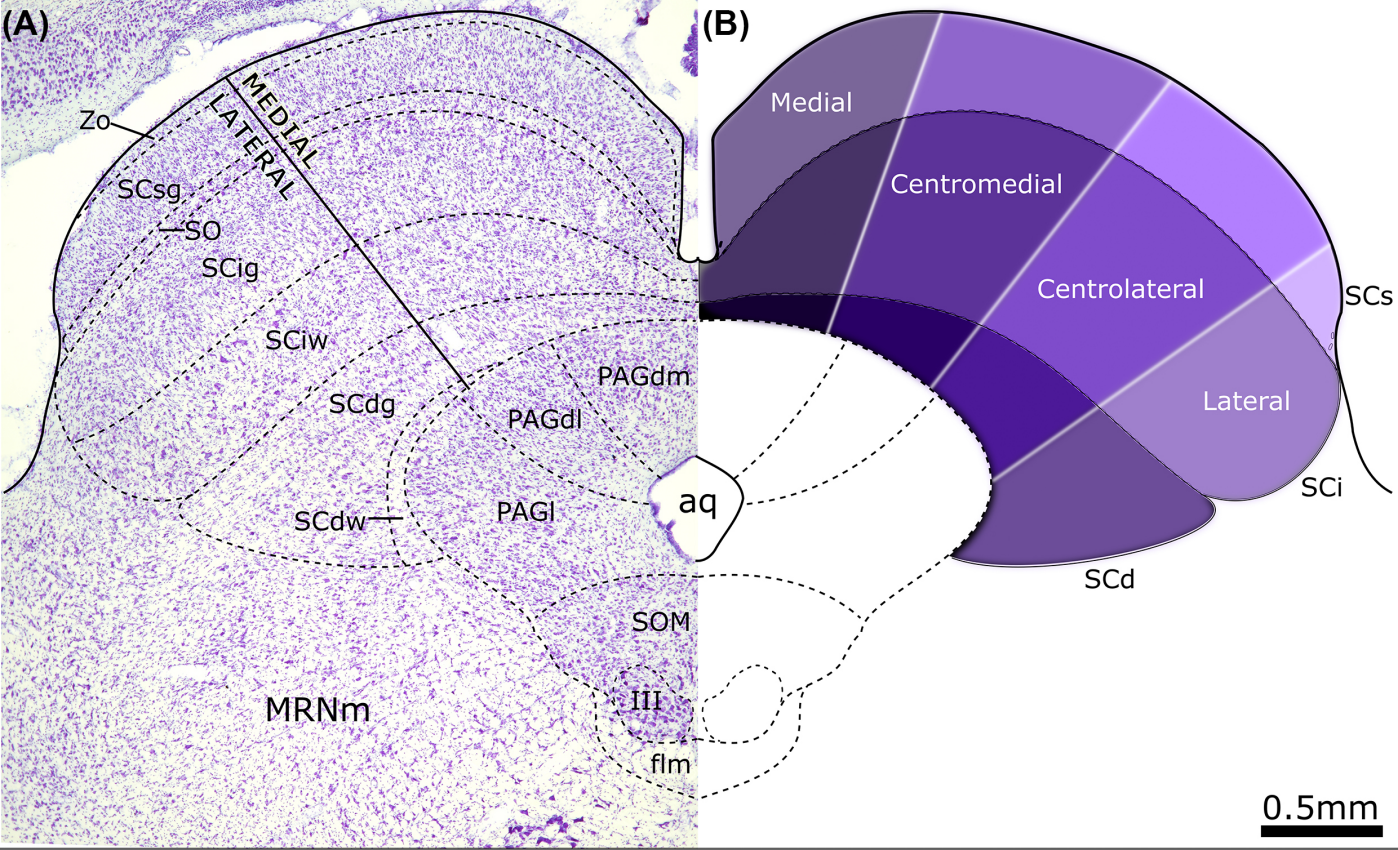
Cytoarchitectonic characteristics and divisions of the mammalian (rat) Superior Colliculus. (**A**) Nissil stained section of the mesencephalic tectum of the rat showing the laminar subdivisions of the SC and the underlying periaqueductal gray. (**B**) Schematic drawing showing a further mediolateral mapping of the SC based on hodological data by [36]. **Abbreviations:** III, Oculomotor nucleus, aq, Aqueduct; flm, medial longitudinal fascicle; MRNm, Medial mesencephalic reticular nucleus, PAG, Periaqueductal gray, PAGdl, dorsolateral PAG; dorsomedial PAG; PAGl, lateral PAG. SCd, Deep layers of the Superior Colliculus, Zo stratum zonale, SCsg superficial stratum griseum, SO stratum opticum, SCig intermediate stratum griseum, SCiw intermediate white layer, SCdg deep stratum griseum, SCdw deep white layer, SCi intermediate superior colliculus. SCs superficial layers of the superior colliculus.

The mammalian SC is organized in layers of distinct cytoarchitectonic and hodological characteristics that consequently lead to distinct functional properties (Fig. **1A**). The collicular layers can be grossly divided into three distinct sections, from dorsal to ventral: superficial (SCs), intermediate (SCi), and deep (SCd) layers (Fig. **1A**, **B**).

The superficial layers are comprised of the *stratum zonale* (Zo), the narrow outermost layer, virtually devoid of cell bodies; the superficial *stratum griseum* (SCsg), which contains most of the cell bodies of the SCs; and the *stratum opticum* (SO), predominantly containing axons, including those arising from the retina, although some cell bodies can also be observed (Fig. **1A**).

Situated directly beneath the SCs are the two layers that compose the SCi. The intermediate *stratum griseum* (SCig), comprised mainly of multipolar neurons, and the intermediate white layer (SCiw), which contains more sparsely distributed cells than the SCig, as well as rostrocaudally aligned fibers and fibers running dorsomedially to ventrolaterally [81].

Finally, the SCd is comprised of the deep *stratum grisseum* (SCgd), which, akin to the SCig, contains multipolar neurons, and the deep stratum album (SGdw), the innermost layer of the SC, which contains fibers that separate the SC from the underlying periaqueductal gray (Fig. **1A**).

Aside from its layered cytoarchitecture, the SC also presents a prominent segregation of inputs and outputs. Afferents from different sensory systems reach different SC layers [80]. Furthermore, there is a clear lateral to medial segregation of inputs targeting the SC, as well as of tectofugal efferents [65, 82]. Broadly speaking, the SC may be divided into medial and lateral parts [65, 82], but there is also clear evidence in mice supporting a more complex mediolateral mapping of the SC that divides it into medial, centromedial, centrolateral, and lateral regions (Fig. **1B**) [36], which will be considered as needed to detail particular aspects of SC hodological relationships.

## 3 HODOLOGICAL CHARACTERISTICS OF THE SUPERIOR COLLICULUS

### 3.1 The Superficial Layers

The superficial layers are exclusively visual (Fig. **1A**, **B**), receiving topographically organized inputs directly from the contralateral retinal ganglion cells [18]. In addition, the SCs receive significant projections arising from the ipsilateral primary visual cortex [36]. These two visual pathways, namely de retino-tectal and cortico-tectal, target different neuronal populations within the SC and present segregated projection patterns to the thalamus [16, 83, 84]. Retino-tectal cell targets are more dorsally distributed within the SCs, and, in turn, send projections mainly directed to the lateral geniculate nucleus (LGN) [85]. On the other hand, neurons receiving cortical afferents are more ventrally distributed throughout the superficial layers of the SC [86]. The primary visual cortical area exclusively targets SCs layers, while the secondary visual areas, including the anterolateral, lateral, anteromedial, and posteromedial, target both the superficial and intermediate layers. The medial and centromedial SCs receive input from all visual cortices and represent the upper central and upper peripheral visual fields [36]. Conversely, the centrolateral SCs receives direct inputs from the primary visual cortical domain that represents the lower central and lower peripheral field and the anteromedial secondary visual area [36]. Note that the SCs region that receives direct inputs from the visual cortex projects to the lateroposterior thalamic nucleus (LP) and the parabigeminal nucleus (PBG) [85, 87-89].

*In vivo* electrophysiological recordings coupled with optogenetics showed that wide-field neurons projecting to the LP appear to be glutamatergic, parvalbumin+, and responsive to small, slow-moving stimuli [90, 91]. In contrast, the more dorsally distributed geniculate-projecting neurons of the SCs are mainly GABAergic and responsive to large, fast-moving, or suddenly appearing stimuli in the visual field [90, 91].

The SCs also contain neurons that appear to increase their firing rates when objects are moving in a preferential direction [90-92]. These cell types present narrow receptive fields (NF cells) and send projections to the PBG and toward the deeper collicular layers [91].

Thus, the superficial layers of the SC are predominantly visual and relay information related to motion. The SCs are involved in providing other networks with visual information and also in directing the control of eye and head movements that orient the animal in relation to visually evoked stimuli. Stimulation of the SC produces gaze shifts, indicating that collicular function is necessary for directing the eyes and orienting the head toward visual stimuli [93, 94]. As an example, rats with SCs lesions show impairment in the orientation reflex when a novel visual stimulus is presented [95]. As mentioned, the SCs do not appear to have direct motor outputs, so the question arises: How are visual signals processed in the superficial SC translated into movements?

### 3.2 Integrating the Superficial and Deep Layers

The different cellular types residing in the SCs (mainly the NF cells) send abundant projections to the deeper collicular layers (SCi and SCd) [88, 96-98]. Both the SCi and SCd harbor output neurons that form the tectofugal motor pathways [20]. The presence of neurons involved in movement control observed within the SCig and SCdg prompted neurophysiologists to refer to these as the motor layers, whereas the superficial layers are frequently referred to as the visuosensory layers [99].

Interactions between the visuosensory and motor layers of the SC have been demonstrated by several anatomical and functional experiments. SCs cells target the SCi neurons directly beneath them [100, 101]. Due to this point-by-point organization, SCi neurons share similar receptive fields with their SCs counterparts [102]. Furthermore, by injecting the anterograde tracer *Phaseolus vulgaris* leucoagglutinin, Rhoades and colleagues (1989) have shown axons from the superficial layers of the SC projecting on all other deep collicular laminae and extending even deeper, with labeled terminals as far as the periaqueductal gray [103].

On the functional side, *in vitro* studies have demonstrated that the excitation of SCs cells evokes monosynaptic or oligosynaptic EPSPs in the SCi [23, 102]. Interestingly, SCi EPSPs are enhanced by the GABA antagonist bicuculine, suggesting a tonic GABAergic system suppressing deeper layer excitation [23], which implies a complex visuomotor integration by an intrinsic collicular network [102].

Beyond the direct interlaminar connections of the SC, other pathways also play a role in the flow of information from superficial to deep layers. Tectothalamic SCs neurons, projecting to either the LP or the LGN, also send collaterals to the SCi. These projections have been observed in several species, including primates [23, 96, 97, 104-106]. Conversely, several studies have observed both excitatory and inhibitory pathways arising from the deeper layers aimed at different neuronal types within the superficial layers. These projections seem to be capable of influencing visual responses by enhancing or suppressing cellular excitability within the visuosensory SCs and may be involved in selecting relevant visual stimuli, spatially-directed movement, or modulating saccadic eye movements, increasing perceptual stability during an action and after training [21, 107-113].

Further complexity is added to information processing within the SC by tectotectal connections. The two superior colliculi are connected by commissural fibers arising from tectotectal neurons. These neurons project to the contralateral colliculus and have been observed in both superficial and deep layers [81]. Interestingly, tectotectal neurons can be GABAergic or glutamatergic [114, 115], and these two populations are similarly distributed within the SC [114, 115]. Several functional roles have been proposed for these inter-collicular projections; however, the exact function of these tectotectal connections is still largely unknown.

In summary, the visual information processed in the superficial layers of the SC can be transformed by SCi and SCd efferents and then further transformed by several intrinsic collicular circuits. Importantly, visuosensory signals are transferred to the multimodal intermediate and deep layers. As discussed below, the deeper layers can integrate these visual signals with other sensory modalities and relay this information to other premotor, motor, and cognitive centers to generate a behavioral action [99].

### 3.3 The Deep Layers

Unlike the purely visual SCs, the intermediate and deep layers of the SC receive integrated multimodal information from the entire brain, cerebellar regions, and sections of the spinal cord. Regarding the visual cortical projections to the SC, as noted above, the intermediate layer of the medial and centromedial SC receives inputs from secondary visual areas, including the anterolateral, lateral, anteromedial, and posteromedial areas, and the centrolateral SC receives inputs from the anteromedial secondary visual area [36]. The centrolateral SC is also targeted by the auditory cortex and frontal-eye field motor domain [36]. In contrast to the more medial zones, the lateral SC does not receive any visual inputs; instead, it receives inputs from all somatic sensorimotor cortical areas. These cortical projections are topographically distributed in a unique somatotopic order. The rostral lateral SC predominantly receives denser inputs from the somatosensory primary cortical field related to the mouth, nose, upper limb, and barrel field [36]. By contrast, the somatosensory primary cortical field related to the lower limb and trunk project more densely to the caudal levels of the lateral SC with extensions into the centrolateral SC. Similarly, all primary motor fields project across the lateral SC, overlapping with their counterpart somatosensory primary cortical inputs [36]. Therefore, the centrolateral SC is distinguished from the lateral SC by receiving convergent visual, auditory, and somatic sensorimotor information.

Furthermore, the deeper SC also receives integrated information from higher-order associations and prefrontal cortical areas. The anterior cingulate cortex, retrosplenial cortex, and posterior parietal cortex heavily project to the lateral portions of the SC. These particular cortical regions integrate spatial, auditory, visual, and somatosensory inputs, thereby relaying integrated multisensory information to the SC [36, 116]. In turn, prefrontal regions such as the infralimbic, prelimbic, and orbitofrontal cortices send projections to the lateral and medial SC [36]. Taken together, these cortical areas constitute a ventromedial cortical subnetwork that may be processing signals regarding navigation, memory, and spatial orientation arising from the dorsal subiculum, as well as interoceptive and emotional components coming from other limbic structures, thus relaying highly integrated information to the deep SC layers [36, 116-119].

Overall, the lateral SCi and SCd are mainly targeted by unimodal and integrated sensory information arising from cortical areas, as well as inputs from motor cortices; the more medial portions of the deep SC integrate information from distinct subnetworks involved in arousal, spatial navigation, and defense [36, 120, 121].

Corroborating the hodological findings, Meredith and Stein (1983) found a population of collicular neurons that are exclusively multisensory, responding to both auditory and visual stimuli in an additive manner. These neurons, however, did not increase electrical activity when either stimulus was presented individually [122]. Another population of neurons was observed to be exclusively unimodal, responding to only one sensory modality, and yet another neuronal population whilst cross-modal stimulation decreases the response when compared to the response recorded during unimodal stimulus presentation [122]. It has been estimated that approximately half of the neurons in the deeper collicular layers of the cat respond to two or more sensory modalities, whereas in primates, that number seems to be less expressive, about 25% [123].

Besides cortical inputs, the medial SC is targeted by the lateral habenular nucleus (LHb) [36, 124]. The LHb is thought to encode negative reward prediction error and, thus, is involved in controlling motivational states and the expression of motivated behaviors. For a review of the structure and function of the habenula [125-130].

In addition, another important input to the SC arises from the ventromedial hypothalamic nucleus (VMH). The medial SCi receives projections from the dorsomedial (VMHdm) and central (VMHc) parts of the VMH. Both the VMHdm and VMHc control innate defensive responses to predators [131-135]. Notably, while the medial SC is targeted by the VMHdm/c, the lateral SC receives input from the ventrolateral portion of the VMH (VMHvl) [36]. Conversely, the ventrolateral VMH is not involved in the expression of defense but rather in behaviors such as approach, reproductive, appetitive, and social defense, and maternal aggression [135-142]. Moreover, the medial, but not the lateral SC, receives projections from the dorsal pre mammillary nucleus (PMD) [82, 143]. The PMD is another important node in the hypothalamic predator-responsive system, that integrates threatening olfactory cues arising from the amygdala with other brain areas related to the expression of defensive responses, as well as aversive memory formation [134].

This medial/lateral input dichotomy also appears to hold for the incerto-tectal pathways. The zona incerta (ZI) makes reciprocal connections with the SC. Interestingly, while the caudal ventral ZI relays vibrissal sensory information to the lateral SC, the medial SC receives input from rostral portions of the ventral ZI, which conveys body-related information [82]. As pointed out by Benavidez *et al.* (2021), projections from the ZI to the medial SCs and SCi support the alignment of visuomotor maps, providing proprioceptive information about the body, whereas the somatosensory nature of the ZI > lateral SC projections are consistent with the placement of the lateral SC within a functional network implicated in appetitive, approach, and orienting behaviors [36].

By analyzing the segregation in afferent patterns between the medial and lateral SC, it should be no surprise that the two regions present distinct functional roles in regulating behavior. The lateral SC has been identified as a critical component of sensory-guided orienting decision-making circuitry [67, 144-148]. During insect hunting, for example, rats with bilateral lesions of the lateral SCi/SCd show impairment in orienting themselves toward moving prey. Lateral SC-lesioned rats also lose the stereotyped sequence of actions normally displayed after capturing the prey, such as holding and manipulating the insects with their forepaws [65]. Furthermore, neuronal activity in the lateral regions of the SC is related to specific movements of the eyes and head, as well as spatially specific shifts of attention and movement selection [149-152]. In addition, unilateral stimulation of the lateral SC prompted eye, whisker, and head movements directed to or away from the contralateral side of collicular stimulation [32, 59, 153]. Interestingly, some studies reporting pursuit-like behavior after lateral SC stimulation also describe the presence of orofacial movements (*i.e*., biting or gnawing) [154]. These results appear to be congruent with the integrative nature of the aforementioned sensory information arriving in the lateral SC [64].

Conversely, stimulation of the medial SC elicits defensive-like behavioral responses, such as flight or freezing, autonomic modulation associated with defense (*i.e*., increase blood pressure and heart rate), as well as increased EEG cortical arousal [145]. By and large, the functional role of the medial SC also appears to be consistent with its above-mentioned inputs.

In contrast to the superficial layers, the SCi and SCd harbor output neurons that send descending projections toward the brain stem and spinal cord, as well as ascending projections to the zona incerta, thalamus, and basal ganglia. Two major descending output paths emerge from the deep layers of the SC. The first follows the predorsal bundle, which, as shown in Fig. (**2**), targets several contralateral pontine and medullary sites [74, 155]. This tract crosses the midline through the ventral tegmental decussation descending medially through the brainstem. The second, as shown in Fig. (**2**), projects ipsilaterally forming the tectopontine and tectobulbar tracts that descend laterally, reaching the dorsal periaqueductal gray, cuneiform nucleus, parabigeminal nucleus, and the capsule of the inferior colliculus, as well as the ventrolateral pontobulbar reticular formation [36, 37, 65, 74, 155, 156].

Consistent with the segregated afferents and functional roles of the SC, in rodents, the crossed descending pathway arises mainly from the lateral SC, thus being responsible for controlling the contralateral oriented movements elicited by its stimulation [30, 32, 37, 56, 65, 74, 156]. Conversely, the uncrossed projections arise mainly from the medial SC, and its efferent pattern, especially to the cuneiform nucleus and dorsal periaqueductal gray, seem to be in agreement with the functional role of the medial SC in organizing defensive behaviors [74].

In a recent study by Isa *et al.* (2020), the authors further dissected the efferent segregated pattern of the crossed and uncrossed projections of the SC, utilizing cre-dependent viral vector constructs to trace and independently stimulate these pathways in mice [74]. The authors showed that optogenetic stimulation of the crossed pathway elicited short-latency stereotyped orienting head and body turns, whereas the stimulation of neurons projecting through the uncrossed pathway prompted long-latency defensive responses [74].

In the same study, in addition to confirming the projection fields for the crossed and uncrossed pathways observed in previous studies utilizing classical tract-tracing techniques [37], the authors observed that both pathways presented crossed/uncrossed collaterals, as well as ascending ipsilateral collaterals, to the midbrain, zona incerta, and thalamus [74]. In addition to the study performed by Isa and colleagues (2002), several other works observed ascending projections from the SCi/SCd to the thalamus, subthalamus, and other midbrain nuclei [65, 157-159].

Regarding the projections to other midbrain areas, the medial SC projects to the dorsal and ventrolateral columns of the periaqueductal grey (PAG), a crucial node in the defense-organizing circuitry [134]; whereas the lateral SC projects to the lateral PAG, which is involved in modulating the motivational drive to hunt and forage [65]. Moreover, both the medial and lateral SC project to the substantia nigra *pars compacta (SNc)* and the ventral tegmental area (VTA). As explained below, these areas generate dopaminergic input to the cortex and ventral striatum and are likely to convey signals representing reward prediction errors used in motor learning processes to promote actions that will maximize future responses [75, 158].

Both the medial and lateral SC project to several thalamic nuclei, from where information about the collicular function, motor planning, and motor output is relayed to the cortex and the basal ganglia [74, 78]. SC information relayed to the cortex *via* the thalamus may modulate general cortical activity [120, 121]. The activation of thalamic regions targeted by the SC increases arousal and behavioral responses to visual threats [160]. Additionally, SC information can provide the cortex with information about the orienting motor plan, such as impending eye movement, so prefrontal cortical areas can coordinate and adapt future behaviors accordingly [161].

The amount of sensory and motivational/affective information being integrated by the SC, as well as the complex motor control exerted by the SC driving adaptive behavior output, most likely require an architecture that accounts for sensory input selection, motor plan, and output selection to properly organize sensorimotor action. Indeed, connections between the SC and the basal ganglia, as well as between the SC and the cerebellum, were identified by several anatomical and functional studies as capable of serving the above-mentioned functions. In the present review, we focus on the interactions between the SC and the basal ganglia. SC-cerebellar loops are outside the scope of the current work and have been reviewed elsewhere [81, 162].

## 4 INTERACTIONS BETWEEN THE SUPERIOR COLLICULUS AND THE BASAL GANGLIA

The ascending projections arising from SC reach the basal ganglia *via* direct connections to its various nuclei or *via* indirect projections to thalamic and subthalamic components. As we shall review, the SC projects to the thalamic groups that covey collicular information to the striatum. The SC also provides direct projections to the subthalamic nucleus [157, 163], representing an extra pathway for the tectal modulation of the basal ganglia [157]. Furthermore, information from the SC may also be relayed to the basal ganglia through midbrain tectonigral and tectotegmental projections directed by SNc and VTA, respectively, influencing the dorsal and ventral (or limbic) divisions of the striatum [164, 165]. On the afferent side, the basal ganglia project back to the deep layers of SC *via* the SNr, which in turn, receives input from the striatum, thus forming a closed SC-thalamic-BG-SC loop [76, 78]. As seen in the cortical interplay with the basal ganglia, this type of closed-loop interaction facilitates the control of appropriate motor behaviors.

### 4.1 The SCs > LP Pathway

The visuosensory superficial layers of the SC send projections to the LP thalamic nucleus (pulvinar in primates). The LP is part of the extrageniculate visual thalamus and sends projections to the extrastriate visual cortex. In addition, projections from the LP also target the striatum (*i.e*., the dorsolateral putamen), which, in turn, provides GABAergic inputs to the SNr, which sends inhibitory projections back to the same region of the SCs [76, 78].

The LP receives dense input arising from the wide-field neurons (WF cells) in the SCs [166]. WF cells mainly respond to small visual stimuli moving in any direction [90, 91] and have been implicated in mediating motion detection in the SC [167]. Interestingly, chemogenetic suppression of wide-field LP-projecting cells while mice were hunting insects impaired rapid prey detection without disrupting other aspects of hunting behavior, such as orienting toward and approaching prey [168].

The projections arising from the LP provide the striatum with visual information relayed through the SCs [169, 170]. Visual information is then conveyed from the striatum to the lateral SNr. In monkeys, neurons harbored in the lateral portions of the SNr were shown to have consistently large spontaneous activity and to decrease or cease their firing rates in response to visual stimuli, as well as during saccadic eye movements [171]. On the other hand, electrophysiological recordings in awake monkeys showed that the caudate neurons located in the regions projecting to the SNr have no spontaneous bursts but rather, increase their firing rates in relation to eye movements [172]. Additionally, electrical stimulation of the saccade-related neurons in the caudate nucleus of monkeys induced inhibition of the substantia nigra saccade-related neurons [173]. The inhibition of the SNr neurons prompted by striatal activation appears to disinhibit neurons of the SC [174]. Thus, the net effect of this loop may release SC neurons from a tonic inhibition allowing for the expression of orienting movements organized in the SC [175].

The functional role of the visuosensory interaction with the basal ganglia has been mostly studied in the context of saccadic eye movements in primates [2]. The LP-mediated loop and its contribution to overall behavioral regulation have only recently begun to be investigated [2, 168, 176-179].

The striatum is not the only significant projection field of LP neurons. Among other targets, the LP also projects to the lateral amygdala (LA) and the lateral extrastriate cortex (LES) [178]. Interestingly, the LP projects to the same sites in the LES that project to the striatum [178]. Thus, the LP can also influence the striatum indirectly *via* projections to the LES, which may or may not be involved in regulating orientation and other aspects of behavior. Moreover, in mice, the SC > LP > LA pathway was implicated in controlling defensive responses elicited by looming stimuli [176]. The SCs excitatory cell population projecting to the LP appears to be involved in the expression of innate visually evoked defensive responses to looming shadows. Looming stimuli in the upper visual field activate the excitatory SCs neurons that send projections to the lateral amygdaloid nucleus relayed through the LP [180], and the activation of the SC > LP > amygdala pathway elicited freezing in response to looming shadows [176, 181]. Indeed, activation of the SC > LP projections elicited freezing responses that were abolished by LA lesions [176]. Importantly, the LP receives not only projections from the SCs, but also ascending collateral projections from the uncrossed (defense-related) pathway neurons residing in the medial SCi/SCd [74]. Thus, whether both the deep and superficial layers contribute equally to the same LP-striatum relayed SC loop or if they constitute two segregated pathways warrants further investigation. Furthermore, it is crucial to investigate whether the projections arising from the SCi/SCd > LP pathway also influence striatal function and to determine their contribution (if any) to orienting behavior.

### 4.2 The SCd > Parafascicular Thalamic Nucleus > BG Pathways

The parafascicular thalamic nucleus (Pf) is the main thalamic target for SC projections and represents the main interface between the SC and the basal ganglia (Figs. **3A**, **B**) [36, 182, 183]. As shown in Fig. (**4**), the four zones of the SC provide a topographic projection to the Pf, where the medial, centromedial, centrolateral, and lateral parts of the SC project to the dorsal, upper-lateral, medio-lateral, and ventrolateral regions of Pf, respectively [36]. In fact, the centrolateral and lateral parts of the SC provide the most extensive projections to the Pf. The Pf, in turn, projects topographically to the striatum, where the dorsomedial (DMS), dorsolateral (DLS), and ventrolateral (VLS) striatum receive projections, respectively, from the dorsal, upper and medio-lateral, and ventrolateral regions of the Pf [36]. This topographical arrangement is particularly helpful to better define the striato-nigral loops projecting back to the SC. As shown in Fig. (**4**), the medial SNr domain receives convergent inputs from the dorsomedial striatum and generates dense projections to the medial and centromedial SC. In contrast, the centrolateral domain of the SNr receives the densest inputs from the dorsolateral striatum and projects specifically to the centrolateral SC. Finally, the lateral SC receives dense inputs from the SNr domain specifically innervated by the ventrolateral striatal domains.

The projection from the SC to Pf may operate in combination with alternative pathways from the SC. The investigation of parallel pathways from the SC to the Pf and ventral zona incerta/posteromedial nucleus of the thalamus (POm) should shed light on how the SC may influence the dorsolateral striatum. The POm does not receive significant direct input from tectal neurons [184]. Interestingly, optogenetic stimulation of the lateral deep SC was shown to elicit a low-latency activation of the Pf nucleus coupled with a prolonged inactivation of POm neurons [185]. This inhibitory effect is due to GABAergic input relayed from the SC to the POm by the ventral zona incerta (ZIv) (Fig. **5**). In turn, the POm projects to the DLS, which is also influenced by excitatory projections from the Pf (Fig. **5**) [184]. It is interesting to note that, unlike the Pf, the POm projection to the striatum seems to be mainly restricted to the DLS [186-188]. In contrast to the Pf, which receives multimodal sensory information from the SCi and SCd, the POm responds to somatosensory input and not significantly to auditory and visual stimuli [184-186, 189]. In rodents, the POm is especially activated by whisker stimulation [184] and also receives direct projections from the trigeminal sensory nucleus. In contrast, somatosensory information reaches Pf through a multisynaptic circuit dependent on integration from deep tectal neurons [190, 191]. Interestingly, POm neurons appear to adapt slowly, continuing to respond even after repetitive whisker stimulation [186, 187, 192]. On the other hand, repeated whisker stimulation tends to hyperpolarize Pf neurons, decreasing the somatosensory input relayed to the DLS [186, 187, 192]. In light of its hodological characteristics and neurophysiological properties, it has been suggested that thalamostriatal projections from the Pf and POm participate in a feed-forward network that regulates the interaction between the SC and DLS (Fig. **5**) [185, 191].

Thus, the SC can inhibit direct somatosensory information reaching the DLS by inhibiting the POm *via* the ZIv, while favoring multisensory information arising from the Pf > DLS pathway. Effectively, this circuit would be capable of shifting orientation and re-directing attention to more salient stimuli in the environment, as well as affecting which motor program is implemented by the DLS.

In contrast to other striatal districts, and much like what was observed for the POm, the DLS predominantly responds to somatosensorial stimuli [191, 192]. In mice, medium spiny cells in the DLS depolarize in response to whisker deflection yet fail to do so in animals presented with visual stimuli [193]. Functionally, the DLS is a crucial node in the basal ganglia circuitry involved in the expression of well-learned behaviors and motor habits displayed in familiar contexts, as well as habit acquisition [194]. Furthermore, the DLS is also involved in the expression of highly stereotyped, repetitive, somatosensory-guided behaviors [191]. In rodents, DLS electrical activity correlates with exploratory behaviors [191]. Also, lesions to the DLS disrupt grooming patterns [195]. Interestingly, this disruption of grooming stereotypy was not observed after lesions to the motor and sensory cortical areas projecting to the DLS [196, 197], suggesting that the motor plan for such behaviors is implemented by the DLS using somatosensory information arising from subcortical regions, possibly the SC or other POm-projecting regions [191].

As mentioned, the Pf receives projections from the SC and sends excitatory projections to virtually all regions of the striatum [182]. Conversely, given its interactions with the ZIv, the SC is capable of suppressing stereotypic and learned behaviors by activating the ZI > POm > DLS pathway (Fig. **5**). Given the SC's crucial role in re-directing attention and orientation, the preferential activation of one of the opposing pathways may represent an essential mechanism for selecting appropriate, adaptive behaviors. As illustrated in Fig. (**5**), either pathway could influence behavioral output by modulating thalamostriatal and thalamocortical sensory outflow. However, the actual behavioral influence exerted by these pathways remains unclear, and further investigation is needed to fully understand their roles [185, 191, 198].

### 4.3 SCd Projections to Other Thalamic Nuclei

In addition to the aforementioned thalamic districts, the deep and intermediate layers of the SC also target other nuclei of the thalamus, which in turn relay tectal information to the basal ganglia and other prosencephalic areas [36, 74, 189, 199]. Thus, apart from the LP and Pf, the SC projects to several intralaminar and midline thalamic groups and to the ventromedial thalamic nucleus (VM). The functional significance of these SC > thalamic pathways is less investigated and warrants further research. Here, we shall consider the hodological characteristics of their possible influence on basal ganglia activity.

#### 4.3.1 SC Projections to the Intralaminar Thalamus

The projections of the SC to the intralaminar thalamic group (including the Pf) are by far the best understood regarding their anatomical characteristics and putative functions [199-201]. First, projections to the central lateral thalamic nucleus (CL) primarily arise from the intermediate layers of the SC [199, 200]. The SCi input to the CL seems to arise from both the medially-distributed neurons of the uncrossed tectofugal pathway, as well as from the laterally distributed, crossed pathway neurons [74]. Furthermore, fluorogold injections in the substantia nigra combined with true blue injections into the CL resulted in a significant number of double-labeled cell bodies in the SCi, suggesting that the SC may simultaneously influence the substantia nigra while sending ascending information to de BG *via* the CL [189]. Of relevance, the CL sends intense projections to the DLS, as well as to cortical areas, especially to the dorsal anterior cingulate cortex and secondary motor cortex [202-204].

The CL, along with the central medial (CM) and paracentral (PC) nuclei, comprise the rostral intralaminar thalamic nuclei [203]. Importantly, there are significant differences between these other rostral intralaminar nuclei regarding their connections with the SC. Despite also receiving projections from the SCi, in contrast with the CL, both the CM and PC receive input mainly from the lateral SCi [199, 200]. Not surprisingly, only modest labeling of ascending collaterals from the uncrossed pathway can be observed in the CM and PC, whereas labeling arising from axon collaterals from the crossed pathway was stronger in these nuclei [74].

Interestingly, the PC pattern of projection upon the cortex resembles that described for the CL. Specifically, the PC sends projections mainly to the dorsal anterior cingulate cortex; likewise, a few fibers originating from the PC could also be observed in the secondary motor cortex [202]. Similarly, the CM also projects to the anterior cingulate cortex, as well as to the primary motor and primary somatosensory cortices [202].

Regarding their striatal targets, in contrast to projections from the CL nucleus, both the PC and CM target the DMS throughout its whole rostrocaudal expanse [202]. Furthermore, both the rostral CM and the PC send projections to the core of the nucleus accumbens (NAc) [202, 204].

In summary, the rostral intralaminar thalamic nuclei provide input to sensorimotor cortical areas and primarily to the dorsal striatum. In addition, these nuclei also provide projections, to some extent, to the limbic districts of the cortex and the ventral striatum.

To date, defining a single function of the rostral intralaminar thalamic nuclei has proven to be a challenging endeavor [203]. Due to their hodological characteristics, these nuclei were implicated in several functions, such as arousal, pain modulation, sensory-motor coordination, and cognition [203]. However, no primary function has been attributed to any of these nuclei. Notably, there is some evidence that the interaction between the SC and the intralaminar thalamus may mediate behavioral action selection, reinforcement learning, and salient event detection [201]; however, the functional role of the SC > intralaminar thalamus is still an open question.

#### 4.3.2 SC Projections to the Midline Thalamic Nuclei

The SC also projects several midline thalamic nuclei [199]. The nucleus reuniens (RE) is an important SC target within the midline thalamus, although projections to other nuclei, such as the paraventricular, rhomboid, and submedius, can also be observed [199].

The RE receives projections mainly from the rostral SC [65, 199, 205]. These projections arise from the uncrossed SCi and SCd, more medially distributed neurons within the SC [74]. Consistent with the functional observations of the medial SC (discussed above), the RE sends significant projections to several subdivisions of the medial prefrontal cortex, namely to the orbitofrontal, prelimbic, infralimbic, and anterior cingulate cortices [202]. Moreover, the RE sends massive projections to the hippocampal formation [202, 206, 207].

Regarding the RE projections to the basal ganglia, the nucleus provides only sparse projections to the medial dorsal striatum [202, 204] and to the caudomedial portion of the NAc [204].

Functionally, the RE has been implicated in several cognitive, affective, and learning processes, as well as in memory formation [203]. Moreover, the RE was observed to harbor cells sensitive to head orientation [208]. Thus, information about orientation and head positioning arising from the SC could be relayed to the hippocampal formation *via* this SC > RE pathway [36, 208].

#### 4.3.3 SC Projections to the Ventromedial Thalamic Nucleus

The VM constitutes another important target for SC projections [65, 199, 200, 207]. These VM-projecting cells are mainly located in the lateral SCi [65, 199, 200, 207] and, to a lesser extent, in the medial SCi [74, 199]. Indeed, PHA-L deposits within the lateral SCi yielded a dense terminal field that covered virtually the entire rostrocaudal extent of the VM [65].

The VM also sends sparse diffuse projections to most of the striatum [207] and the dorsolateral part of the NAc [204, 207]. Considering the cortical projections, the VM provides a dense projection to the most superficial part of layer I, extending over almost the entire neocortex [207], influencing, perhaps, general arousal levels. Moreover, the VM shares reciprocal connections with motor somatic and sensorimotor cortical areas representing the trunk and lower limbs [36, 202, 207]. These projections may form a somatotopically organized cortico-tecto-thalamic loop [36, 202, 207]. Notably, the bulk of the SC > VM projections arise from the crossed pathway [74, 209]. Thus, in sending motor commands to the brainstem and spinal cord, the SC could convey an efferent copy of this motor command to superior motor centers *via* the VM [65].

### 4.4 The SC > Subthalamic Nucleus Pathways

The subthalamic nucleus (STN) is an important entry point to the basal ganglia circuitry. Two putative pathways allow for tectal modulation of STN function, which may also influence BG output. First, the parafascicular nucleus of the thalamus (Pf), which, as previously discussed, is heavily targeted by the SC, sends projections to the STN [210-212]. Second, the lateral SC sends direct projections to the STN (hyper-direct pathway) [157, 163]. Since the STN then projects to SNr and internal globus pallidus, these pathways allow for the tectal modulation of the basal ganglia outflow [157].

A significant body of evidence implicates the STN in regulating reward, emotional processing, motivated behaviors, and behavioral switching [213-218]. Additionally, the STN is responsive to the sensory stimuli of several modalities, including auditory, visual, and nociceptive [219-221]. The sensorimotor cortical projections that target the STN are somatotopically organized, providing the STN with a motor map of the body, eyes, and limbs [222]. Interestingly, cortical ablation compromises the STN’s response to sensory stimuli [221].

Beyond cortical interactions, the STN receives sensory information from brainstem regions. As previously mentioned, the lateral SCi/SCd sends information to the STN either directly or indirectly through Pf projections, providing the nucleus with multi-modal sensory information. Thus, the STN appears to be capable of processing sensory information arising from the cerebral cortex and SC simultaneously, allowing for cortical-BG and SC-BG interactions [223].

In recent years, accumulated evidence has indicated that the subcortical-basal ganglia interactions mediated by the STN are organized in parallel, possibly closed loops [78]. Regarding the tectum, an SC > STN > SNr > SC loop has been described in several anatomical and functional studies [78, 157, 163, 220, 224, 225].

Interestingly, STN neurons respond to visual and auditory stimuli with short latencies [223, 226]; however, the STN does not receive direct input from the visual cortex and, consequently, from the retina > LGN pathway [227, 228]. Rather, the fast response to visual input (and presumably auditory as well) arises from the SC [157]. In fact, disinhibition of the SC by intracollicular injections of the GABAa antagonist bicuculine increased the response to visual stimuli of neurons in the STN [157]. Moreover, increased STN responsiveness to visual stimuli was not observed after disinhibiting the medial SC or the primary visual cortex [157]. Thus, the lateral SC provides short-latency input to the STN, which conveys rapid, though somewhat crude, visual information. This input may quickly modify, *via* the STN, the behavioral programs executed by the basal ganglia [34, 122, 157].

Experimental data have also indicated that the STN is an essential node for basal ganglia control of neural and behavioral inhibition. In rats, lesions to the STN could not inhibit impulsive and preservative operant responses even when operant behavior presented a low probability of reward [229]. Moreover, stimulation of the STN inhibits saccade generation by increasing the tonic inhibition of the SC elicited by the SNr (Fig. **6**). Conversely, SNr inhibition removes the GABAergic tonus on the SC, releasing it to favor saccadic eye movements [216, 230].

STN neurons seem to adapt to repeated sensory stimuli, diminishing their response after the stimulus has lost salience [223]. Thus, novel or unexpected environmental cues are likely to activate the STN hyper-direct pathway from the SC, which interrupts ongoing behavior in favor of more adaptive behavioral responses [223]. Importantly, through its complex physiological properties, environmental cues become less likely to activate the STN pathway and, therefore, less likely to change the behavioral output [223]. As pointed out by Al Tannir and colleagues (2022), it would not be adaptive for less salient, repeated stimuli from the environment to interrupt ongoing behaviors. Thus, the characteristics of the STN neuronal response indicate that the impact of sensory inputs coming from the SC can be modulated to favor the selection of particular behaviors [223].

In summary, the STN provides the basal ganglia circuitry with short-latency sensory input coming from the tectum and is capable of modulating behavioral output by selecting relevant sensory information arising from both the tectum and prefrontal cortical areas.

Beyond the hyper-direct pathway, the STN receives projections from the Pf [210, 228, 231], which in turn is targeted by tectal projections [65, 199] (Fig. **6**). Much like the hyper-direct pathway, this pathway also bypasses the striatum, however, due to an extra synapse in the Pf, it was termed the “super-direct” pathway [212]. Moreover, rather than relying on direct corticomotor/tectal projections to the STN, this pathway seems to arise exclusively from Pf inputs from sensory and associative areas of the cortex and brainstem (*i.e*., from the SC) [212].

Although this pathway has only begun to be explored, the Pf > STN may constitute an important means for the interaction between SC and BG structures. The Pf nucleus harbors a heterogenic neuronal population. Pf neurons projecting to the striatum and the STN do not seem to overlap [211, 212, 231]. Interestingly, STN-projecting Pf neurons appear to partially overlap with the population providing afferents to the SNr [211]. Furthermore, electrophysiological and morphological studies identified at least two different populations present in the Pf [232, 233].

Due to its recent conceptual development, the functional role of the super-direct Pf > STN pathway is still poorly understood. Nevertheless, Watson *et al.* (2021) observed that the optogenetic stimulation of the Pf > STN, but not the Pf > striatal projections, elicited movement initiation in mice. Thus, the Pf can influence motor behavior (*i.e*., orienting movements) independent of the striatum by modulating locomotor regions *via* the STN [212]. In addition, optogenetic stimulation of STN-projecting neurons yielded a complex electrical response pattern in the SNr, notably early excitation followed by short-term inhibition [211]. This pattern was not observed when striatal-projecting Pf neurons were stimulated. In fact, activation of the Pf > striatum pathway prompted the inhibition of SNr neurons [211].

It is important to note that the role of these different pathways in regulating behavior, as well as the interplay of the super-direct pathway and other BG structures, has yet to be elucidated. Moreover, in theory, both the hyper-direct and super-direct pathways might act on the action selection of salient stimuli by integrating SC sensory input *via* the Pf > STN. It would be interesting to investigate the actual contribution of each pathway to motor control, as well as the complex information processing that could possibly arise from the interplay between both pathways.

### 4.5 The SC > Substantia Nigra *pars compacta*/Ventral Tegmental Area Pathway

As discussed, the SC may influence the basal ganglia through indirect thalamic/subthalamic pathways, as well as through direct projections to the STN. In addition, other direct pathways from the SC modulate BG function. The intermediate and deep layers of the SC send direct projections to the dopaminergic neurons of the SNc [75, 158, 159, 189]. Moreover, neurons of SCi and SCd also contact the dopaminergic neurons of the VTA [75, 159, 234]. Interestingly, the SC also sends projections to other dopaminergic cell groups (DA) in the brainstem (*i.e*., the lateral dorsal tegmental nucleus, parabrachial nucleus, and dorsal raphe nucleus). [78].

Although the functional significance of these specific SC > DA projections is still debated, these mesencephalic dopaminergic neurons respond to unexpected, salient events [235, 236]. Furthermore, DA projections arising from these areas (*i.e*., the SNc) modulate basal ganglia function, for example, by activating D1 (excitatory) and D2 (inhibitory) dopaminergic receptor subtypes in the striatum, associated with the direct and indirect pathways, respectively [237, 238]. Thus, dopaminergic input to the striatum inhibits the indirect pathway and activates the direct pathway, ultimately facilitating movement [237-239]. (Grillner and Robertson, 2016)

In terms of information, SC projections provide the ascending dopaminergic system with sensory input. In turn, DA neurons supply the basal ganglia with phasic, short-latency signals that support shifts in attention and learning through reinforcement, maximizing future responses in the face of a novel, biologically-relevant environmental stimuli [240]. In fact, intense sensory stimuli, sudden stimuli, or stimuli associated with reward prompt the increased firing of dopaminergic neurons [235, 236, 241, 242]. This increase in DA neurons’ activity is characterized by particularly short latencies after stimulus presentation, as well as short bursts in activity. Interestingly, recordings from DA neurons show that these cells rapidly habituate once a novel sensory stimulus is presented in repetition and becomes familiar without reinforcement [235, 243-245].

In this regard, the SC can provide both the SNc and the VTA with early extra geniculate visual input causing DA neurons in these regions to fire before visual processing is completed by the retino-thalamocortical pathway [189, 246]. Indeed, the latency of visuocortical activity is about 80-100 ms after a stimulus, whereas the DA latency to respond is lower, around 70 ms [240]. Moreover, after disinhibition of the SC in anesthetized rats by intratectal bicuculine injection, dopaminergic neurons in the mesencephalic tegmentum become responsive to visual stimuli [246]. Conversely, this effect on DA neurons was not observed after disinhibition of the visual cortex [246]. Thus, the SC is an important detector of visual stimuli that can directly influence dopaminergic modulation of the basal ganglia outflow.

Neurons from both the SCi and SCd send direct projections to the SNc [158]. These neurons interact with tyrosine hydroxylase-positive (dopaminergic) and -negative neurons in the SNc [158, 234]. Topographically, the lateral portions of the SNc receive more intense projections from the lateral SC, whereas the medial SNc receives afferents from the more medial regions of the SC. Furthermore, a portion of tectonigral cells also sends collaterals to intralaminar thalamic nuclei (including the parafascicular, central medial, and central lateral), as well as to the ventromedial nucleus [189]. Consequently, in addition to the DA projections from the SNc, short-latency visual input from the SC can reach the striatum *via* intralaminar thalamic relays. Interestingly, visual stimuli promptly reach the striatum concurrent with, or shortly after, phasic dopaminergic discharges [172, 240]. Notably, the SC visual responses linked to DA activation are modulated by inputs from the visual cortex, where aspiration of the ipsilateral visual cortex prompted a reduction in visually evoked potentials in both the SC and SNc, and this reduction was reversed by bicuculine-induced tectal disinhibition [158]. This leads to the idea that cortical input constitutes an important driver of tectal excitatory tone.

The SC also sends significant projections to the VTA [75, 234, 246]. Similarly to what was discussed for the SNc, the VTA also harbors dopaminergic neurons that are known to increase their firing rate with short latencies in response to visual stimulation [243, 244, 246]. Also, disinhibition of the SC in anesthetized rats elicited an increase in burst activity in VTA neurons [234, 246]. Furthermore, VTA activity is related to salient unexpected events and rewards [235, 240, 243, 247, 248]. Anatomically, an important distinction between the VTA and SNc is that while the SNc receives input from more lateral distributed neurons in the SC, VTA input mainly arises from the medial intermediate layers of the SC [158]. Moreover, DA VTA neurons project preferentially to the ventral striatum, namely NAc, in addition to the ventromedial striatum, amygdala, septum, and frontal cortex. In contrast, as previously mentioned, DA SNc neurons mainly provide input to the dorsal striatum [164, 165, 249].

In addition to relaying short-latency visual information to the midbrain DA system, virtually all sensory modalities studied concerning the interactions between SC and the dopaminergic system appear to be transmitted to DA targets with shorter latencies than the cortical > DA input [240]. Strikingly, the sensory information flow to the DA system can occur independent of the cortical activity or be potentialized by the SC. For example, electrical stimulation of the somatosensory barrel cortex of urethane-anesthetized rats elicited phasic activation in the SC that does not prompt a response in midbrain DA neurons [250]. However, after SC disinhibition, barrel cortex stimulation increased activity in the SC, enhancing DA neurons’ response as well [250]. Also, visually evoked potentials can be recorded in DA neurons even in the absence of the visual cortex. Perhaps not surprisingly, DA potentials were abolished after the ablation of the visuosensory layers of the SC [158]. These examples seem to reinforce the interpretation that the SC is the principal source of sensory input to the dopaminergic system and, therefore, is critical for detecting novel salient events, whether they be potentially aversive or rewarding.

Through its anatomical SNc/VTA connections and functional properties discussed above, the SC is in a privileged position to modulate DA function and, consequently, its interactions with the dorsal and ventral BG. Dopaminergic outflow to the dorsal and ventral striatum favors learning and decision-making by reinforcing actions and movements that may result in an unexpected reward or avoidance of aversive stimuli [251]. Therefore, the SC provides the DA system with short-latency, pre-attentive sensory information that allows for the adaptive VTA/SNc > BG interaction. Furthermore, these novel, unpredictive stimuli evoke gaze, attention shifts, and defensive responses, which are also organized by the SC and may increase the sensory information reaching dopaminergic neurons [240, 252].

### 4.6 Possible Implications for Pathology

Impairment in basal ganglia circuitry’s function is implicated in the pathophysiology of several aspects of motor disorders such as Parkinson’s disease, Huntington’s disease and dystonia [239, 253]. Due to the close interactions between the SC, basal ganglia, and dopaminergic system, researchers have investigated functional changes in the SC-BG loops in both human patients and animal models, with a particular emphasis on Parkinson's disease. [224, 254-256].

In Parkinson’s disease (PD), the loss of neurons in the SNc due to neurodegenerative processes leads to striatal dopaminergic depletion. The loss of DA in the striatum shifts the balance of the striatopallidal activity in favor of the indirect (Striatum > External globus pallidus > STN > SNr > thalamus) pathway. This increases thalamocortical inhibition, thus causing deficits in movement execution and initiation [253, 257].

The motor hallmarks of parkinsonian syndrome are slow and limited limb movement (akinesia or bradykinesia) and increased muscle tone (rigidity), often accompanied by tremors when at rest. Another group of movement disorders observed in parkinsonian patients are dyskinesias and hyperkinesias, which involve an excessive and involuntary muscular activity that interferes with voluntary motor commands and disrupts normal intended actions of the limbs [253, 254].

Patients with PD also present marked impairments that are associated with disruption of SC function, such as deficits in eye movements, including hyperexcitable blinking reflexes [258-260] and abnormal memory-guided and visually guided saccades [255, 261-263] (for a review see: [259]). Accordingly, in a recent fMRI luminescence activation study, Moro and colleagues (2020) showed early hypersensitivity to low luminance and abnormal blood oxygenation in the superior colliculus of 22 patients recently diagnosed with Parkinson’s disease [254]. Thus, it has been suggested that oculomotor deficits and changes in tectal activity may be used as biomarkers for detecting early onset PD [256, 257].

Considering the SC-BG pathways described above (Fig. **4**), it is hypothesized that the SC may be inhibited by an overactive SNr (due to a diminished striatal GABAergic input) in PD patients, thus suppressing or disrupting the oculomotor behavior organized by the SC. In line with this hypothesis, Basso and Liu (2007) showed that electrical stimulation of the SNr reduced SC activity and influenced the generation of Saccades in rhesus monkeys [255, 264].

Likewise, SC function has also been investigated in murine models of PD [212, 224]. In anesthetized rats, dopaminergic denervation by SNc injections of 6OH-Dopamine, increased both SC and STN responsiveness to visual stimulation [224]. The SC and STN-enhanced activity could possibly explain the difficulty in inhibiting reflexive saccades observed in PD patients [255, 259, 265]. Furthermore, STN stimulation was capable of restoring natural movements in mice after bilateral DA depletion [212]. It is important to note that the mechanisms and the roles of the SC and STN on limb movement and saccade control, especially saccade inhibition, are not yet completely understood. However, several recent studies investigating this circuitry in both PD patients and animal models were published, thus advancing our current comprehension of this topic [212, 224, 232, 254, 262, 266-268].

**Fig. (2):**
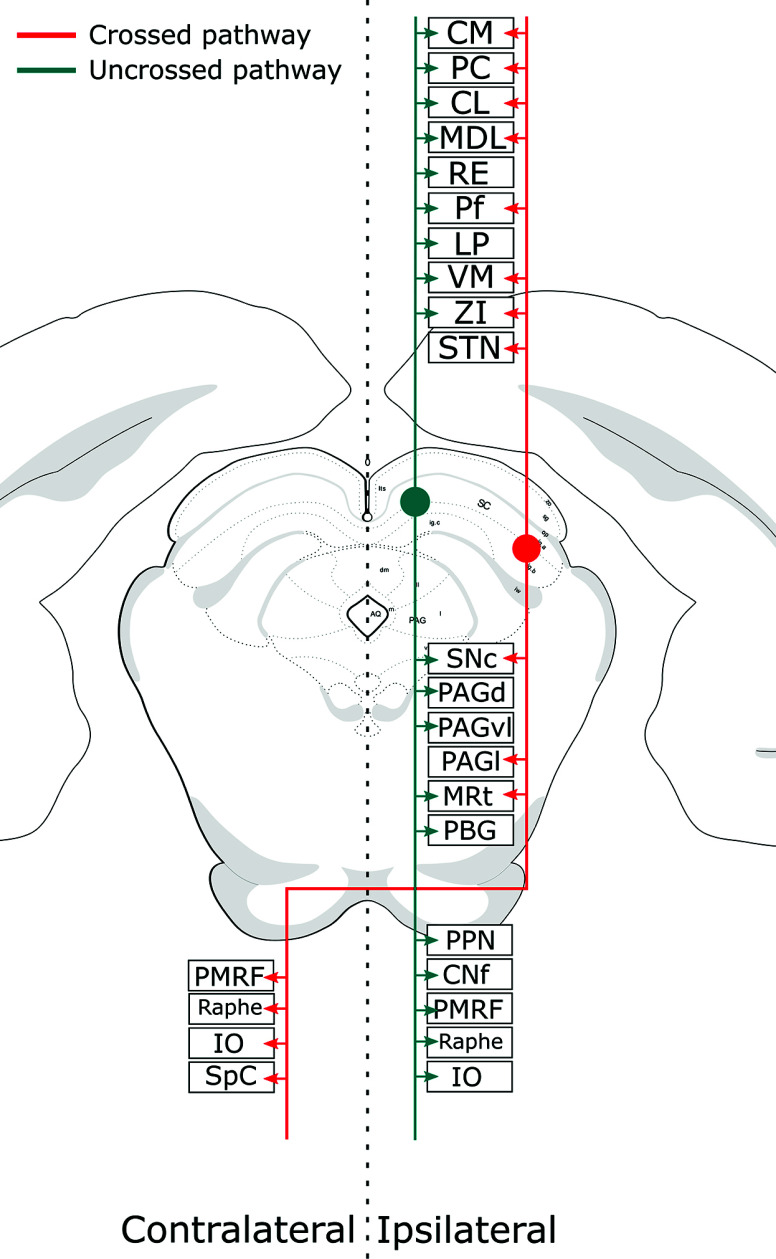
Efferent projections of the Superior Colliculus. Schematic drawing of a coronal section through the mouse midbrain showing (in red) the crossed descending pathway emerging from the lateral SC and the uncrossed descending pathway (in blue) stemming from the medial SC. Schematic drawing also shows the ascending collaterals to the thalamus/Subthalamus of each pathway. **Abbreviations:** CL central lateral, CM central medial, CNf cuneiforme nucleus, IO inferior olivary nucleus, LP lateroposterior, MDL lateral mediodorsal, MRt mesencephalic reticular formation, PAGd dorsal periaqueductal grey, PAGl lateral periaquedutal grey, PAGvl ventrolateral periaquedutal grey, PBG parabigeminal nucleus, PC paracentral, Pf parafascicular nucleus, PMRF medial ponto-medullary reticular formation, PPN pedunculopontine nucleus, RE nucleus reuniens, SNc substantia nigra pars compacta, SpC spinal cord, STN subthalamic nucleus, VM ventromedial, ZI zona incerta.

**Fig. (3):**
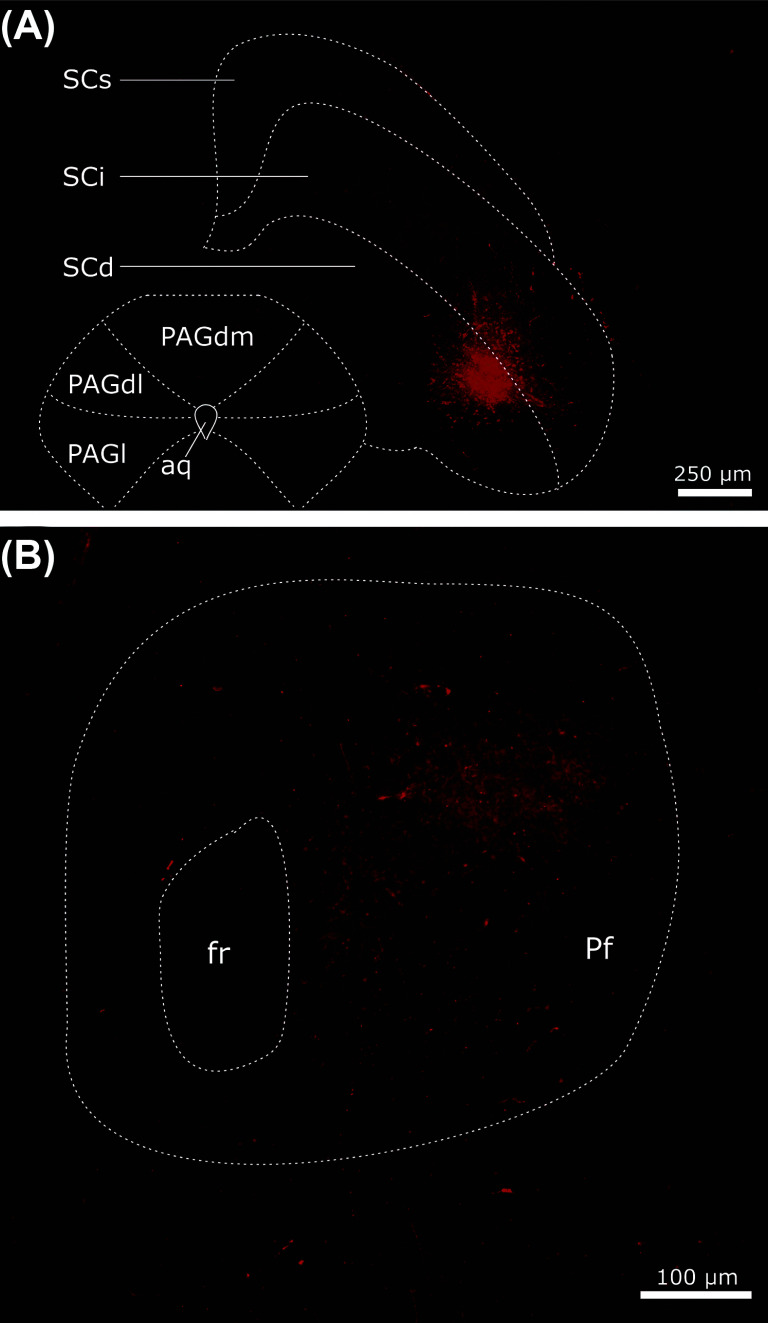
Efferent projections from the lateral superior colliculus to the Parafascicular nucleus of the thalamus. (**A**) Coronal section through the SC of a mouse showing iontophoretic deposit of the anterograde tracer FluoroRuby in the Centrolateral/lateral deep layers of the SC. FluoroRuby deposits yielded consistently labeled fibers in the Pf nucleus as shown in (**B**). **Abbreviations:** Aq aqueduct, Fr retroflex fasciculus, PAGdl dorsolateral periaqueductal grey, PAGdm dorsomedial periaqueductal grey, PAGl lateral periaqueductal grey, Pf parafascicular nucleus, SCd deep layers of the superior colliculus, Sci intermediate superior colliculus, SCs superficial layers of the superior colliculus.

**Fig. (4):**
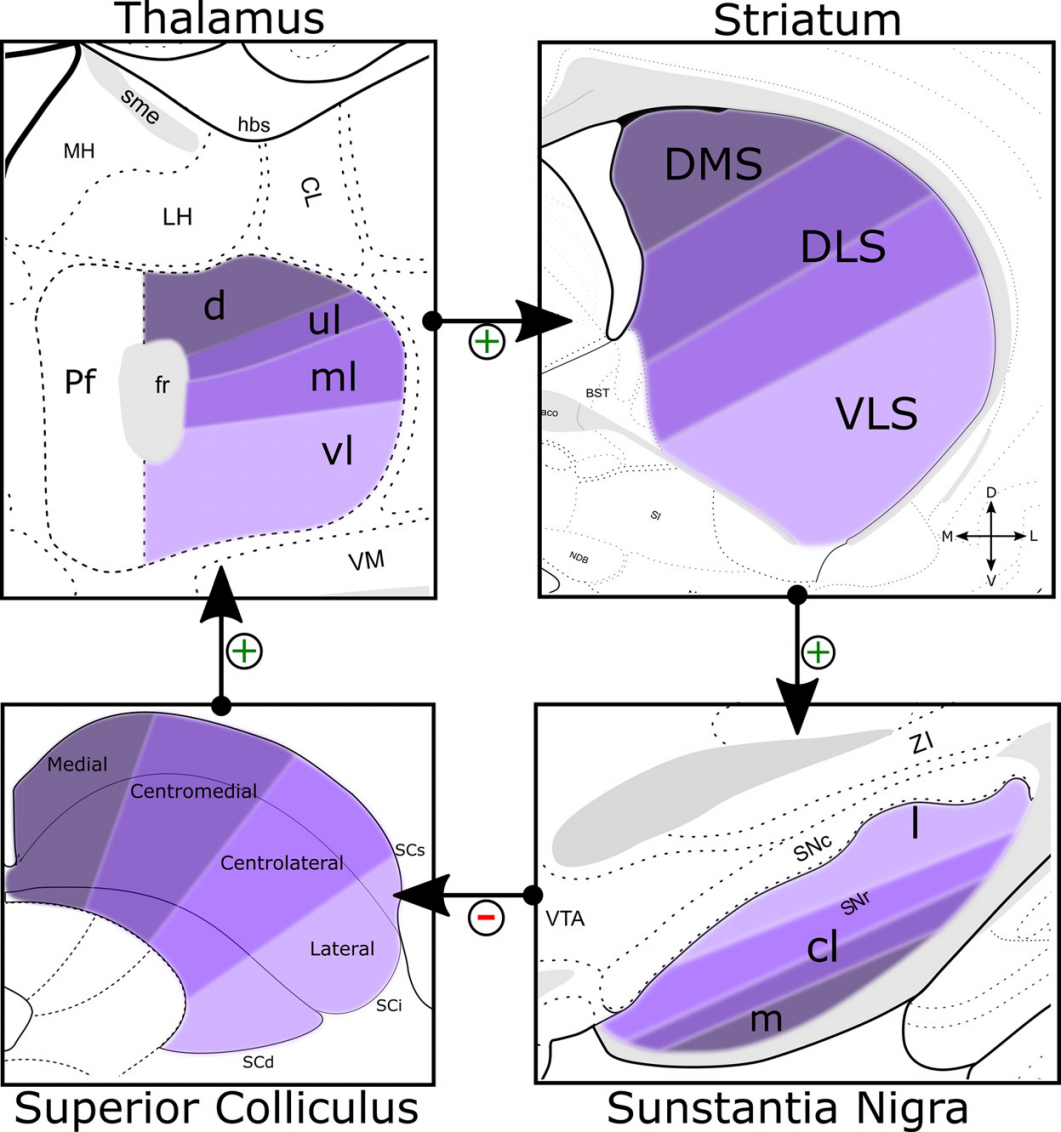
Schematic drawing of different regions of the mouse brain showing the SC>Pf>Striatum>SNr loop. Color match represent overlapping projection domains between the SC, Parafascicular nucleus, Striatum and Substantia nigra. (+) denote Excitatory projections. (–) Denote inhibitory projections. Figure based on experimental data by Benavidez *et al*. (2021) [36]. **Abbreviations:** CL central lateral nucleus of the thalamus, cl central lateral SNr, d dorsal Pf, DLS dorsolateral striatum, DMS dorsomedial striatum, Fr retroflex fasciculus, l lateral SNr, LH lateral habenula, m medial SNr, MH medial habenula, ml medio-lateral, Pf parafascicular nucleus, SCd deep layers of the superior colliculus, Sci intermediate layers of the superior colliculus, SCs superior layers of the superior colliculus, SNc substantia nigra pars compacta, SNr substantia nigra pars reticulata, ul upper lateral, vl ventrolateral, VLS ventrolateral striatum, VTA ventral tegmental area, ZI zona incerta.

**Fig. (5):**
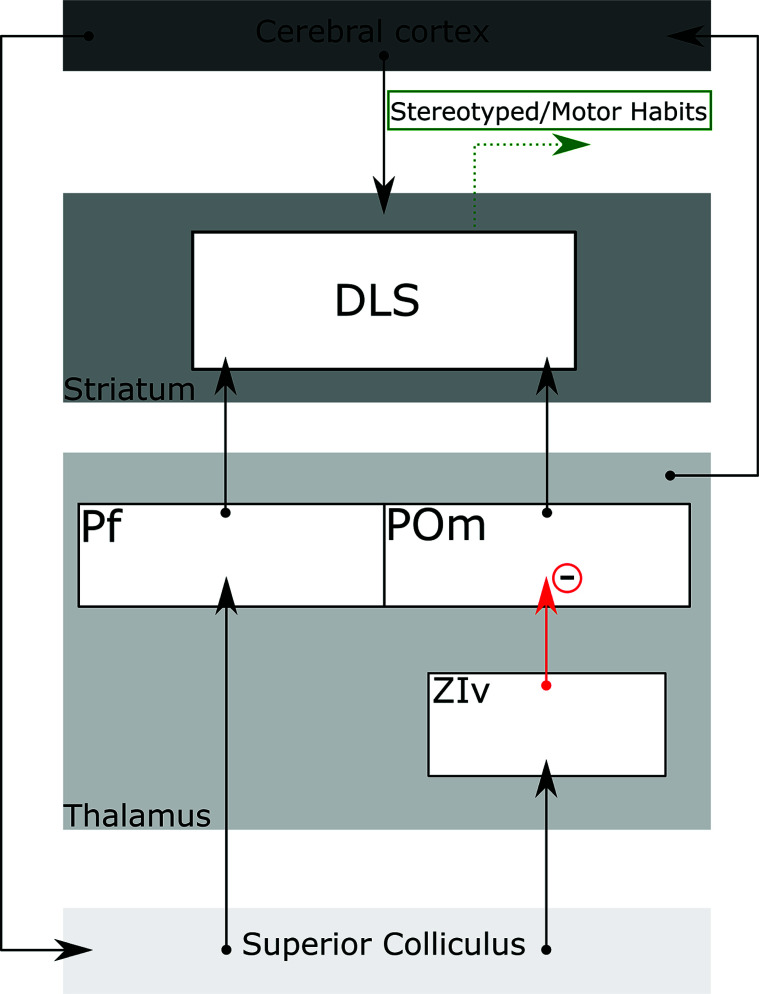
Schematic drawing of two pathways linking the SC and the DLS, The SC>Pf pathway and SC>ZIv>POm pathway. (–) and red arrows denote inhibitory projections. **Abbreviations:** DLS dorsolateral striatum, Pf parafascicular nucleus, POm posteromedial thalamic nucleus, ZIv ventral zona incerta.

**Fig. (6):**
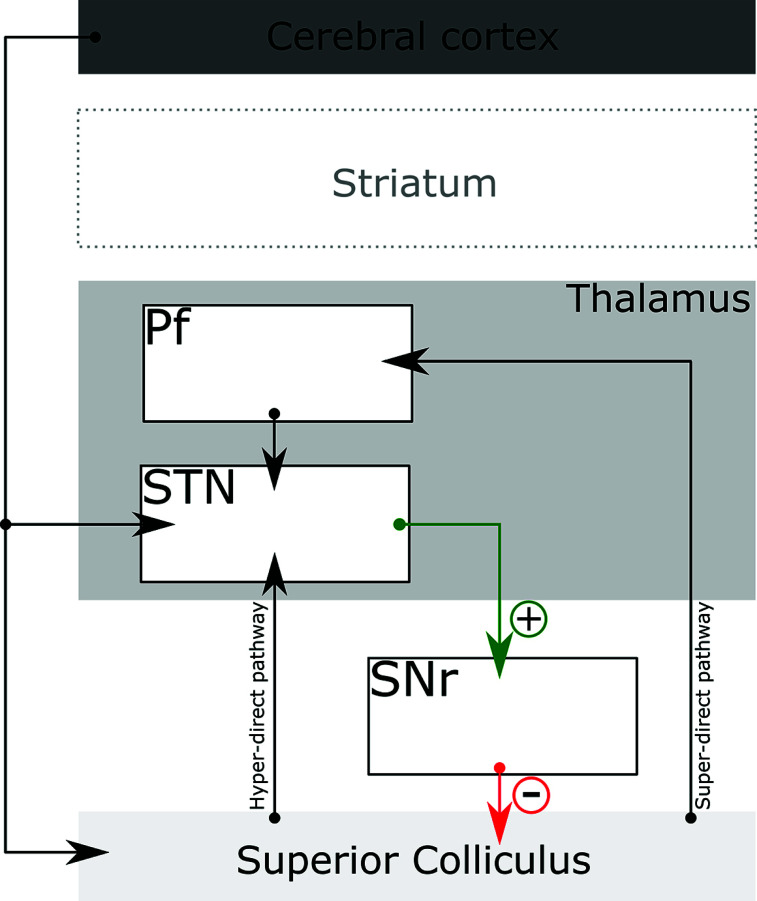
Schematic drawing of the hyper-direct and super-direct SC>STN interactions. (+) and green arrow denotes excitatory projections. (–) and the red arrow denotes inhibitory projections. **Abbreviations:** Pf parafascicular nucleus, SNr substantia nigra pars reticulata, STN subthalamic nucleus.

It has also been argued that other pathways passing through the basal ganglia to subcortical sites, including the SC, may be involved in limb and eye movement impairments observed in PD [255-257, 268]. As an example, deep brain stimulation (DBS) of the ZI, which makes direct and indirect connections with the SC (Fig. **2**; Fig. **5**) ameliorates bradykinesia and tremors in PD patients [269].

There is also evidence to suggest that the motor symptoms of Parkinson's disease may be associated with the cholinergic neurons of the pedunculopontine nucleus (PPN), independently of dopamine depletion. Interestingly, both the superior colliculus (SC) (as shown in Fig. **2**) and subthalamic nucleus (STN) have reciprocal connections with the PPN [257]. One potential mechanism through which deep brain stimulation (DBS) of the STN may improve Parkinsonian symptoms is by enhancing cholinergic activity *via* stimulation of the PPN [257]. However, further investigation is required to determine whether this is a valid explanation for the modulatory effect on SC function in Parkinson's disease patients.

Finally, dysfunction of the limbic and associative BG-cortical loops, as well as impairments of SC-BG loops, have also been thought to be involved in the genesis of non-motor/ affective symptoms that accompany these motor disorders. In a similar fashion, changes in dopamine in these circuits are often associated with changes in mood, reward perception, and other neuropsychiatric symptoms [175, 251, 253, 270, 271]. It has been proposed that symptoms such as anxiety, apathy, and impulsiveness associated with PD or with its pharmacological treatment, might be linked with dysfunction or sensory information flow relayed by the SC to thalamic and amygdalar nuclei [272, 273].

In summary, the involvement of the SC in PD is becoming increasingly apparent, and it appears to be related to the dysfunction of the basal ganglia and other structures involved in motor control. Further research is needed to fully understand the contribution of the SC to the motor and non-motor symptoms of PD and to develop more effective treatment options for this debilitating disease.

## CONCLUSION

The SC presents a well-developed structure that plays a prominent role in visual-guided behaviors. In birds and primates, where visual information is the predominant sensory modality, the SC presents a more well-developed structure.

The complex intrinsic connections between the SC’s laminae and tecto-tectal projections allow for the construction of spatially aligned visual-multisensory maps of the surrounding environment. These multi-modal sensory maps then contribute to the diverse functions of the SC.

The SC is involved in the processing and integrating of a plethora of sensory information, ultimately influencing motor behavior. Since the SC can organize and modulate motor functions, it is not surprising that the SC coordinates its activity with several basal ganglia networks. In this regard, it is relevant that cortical projection neurons generate collateral projections to innervate both striatal and tectal targets.

The ascending projections arising from the SC reach the basal ganglia *via* direct connections to its various nuclei, including the midbrain dopaminergic cell groups and the subthalamic nucleus, or *via* indirect projections to thalamic and subthalamic components that influence the striatum.

Functional studies revealed that through thalamic paths, the SC provides visual information to the striatum, where electrophysiological recordings showed that striatal neurons increase their firing rates in relation to eye movements. Moreover, SC > thalamic > striatal pathways can shift orientation and re-direct attention to more salient stimuli in the environment, as well as affect the striatum's motor program. Since the SC plays an important role in re-directing attention and orientation, SC thalamostriatal outflow may constitute an essential mechanism by which the SC could favor the expression of adaptive behaviors.

However, this investigation is still in its infancy, and further studies are needed to expand our knowledge of the SC influences in the striatum *via* several thalamic paths.

In addition, connections from the striatum to the SNr form re-entrant closed-feedback loops to the SC that allow for adaptive and smooth collicular-driven/modulated actions, mediated through outputs aimed toward the reticular formation and spinal cord.

SC projections also provide the ascending dopaminergic system with sensory input. In turn, DA neurons supply the basal ganglia with phasic short-latency signals that support shifts in attention and learning through reinforcement, maximizing future responses in the face of a novel, biologically-relevant environmental stimuli.

Finally, the SC targets the subthalamic nucleus (STN) through both a hyper-direct pathway (*i.e*., direct SC > STN projections) and a super-direct pathway (*i.e*., SC > STN projections mediated through the parafascicular thalamic nucleus). Novel or unexpected environmental cues likely activate the STN pathways from the SC, which interrupts ongoing behavior in favor of other more adaptive behavioral responses. At this point, a great deal remains unknown about how the hyper-direct and super-direct pathways from the SC might be able to act on the action selection of salient stimuli. It would be interesting to investigate the actual contribution of each pathway to motor control and the complex information processing that may arise from the interplay between these pathways.

## LIST OF ABBREVIATIONS

CM: Central Medial
DLS: Dorsolateral
DMS: Dorsomedial
LA: Lateral Amygdala
LES: Lateral Extrastriate Cortex
LGN: Lateral Geniculate Nucleus
NAc: Nucleus Accumbens
PAG: Periaqueductal Grey
PC: Paracentral
PD: Parkinson’s Disease
SC: Superior Colliculus
SNr: Substantia Nigra
STN: Subthalamic Nucleus
VLS: Ventrolateral
VTA: Ventral Tegmental Area
